# Metabolic labour division trade-offs in denitrifying microbiomes

**DOI:** 10.1093/ismejo/wraf020

**Published:** 2025-02-04

**Authors:** Nina Roothans, Mark C M van Loosdrecht, Michele Laureni

**Affiliations:** Department of Biotechnology, Delft University of Technology, van der Maasweg 9, Delft 2629 HZ, the Netherlands; Department of Biotechnology, Delft University of Technology, van der Maasweg 9, Delft 2629 HZ, the Netherlands; Center for Microbial Communities, Department of Chemistry and Bioscience, Aalborg University, Fredrik Bajers Vej 7K, Aalborg East 9220, Denmark; Department of Biotechnology, Delft University of Technology, van der Maasweg 9, Delft 2629 HZ, the Netherlands; Department of Water Management, Delft University of Technology, Stevinweg 1, Delft 2628 CN, the Netherlands

**Keywords:** denitrification, labour division, genome-resolved metagenomics, trade-off

## Abstract

Division of metabolic labour is a defining trait of natural and engineered microbiomes. Denitrification—the stepwise reduction of nitrate and nitrite to nitrogenous gases—is inherently modular, catalysed either by a single microorganism (termed complete denitrifier) or by consortia of partial denitrifiers. Despite the pivotal role of denitrification in biogeochemical cycles and environmental biotechnologies, the ecological factors selecting for complete versus partial denitrifiers remain poorly understood. In this perspective, we critically review over 1500 published metagenome-assembled genomes of denitrifiers from diverse and globally relevant ecosystems. Our findings highlight the widespread occurrence of labour division and the dominance of partial denitrifiers in complex ecosystems, contrasting with the prevalence of complete denitrifiers only in simple laboratory cultures. We challenge current labour division theories centred around catabolic pathways, and discuss their limits in explaining the observed niche partitioning. Instead, we propose that labour division benefits partial denitrifiers by minimising resource allocation to denitrification, enabling broader metabolic adaptability to oligotrophic and dynamic environments. Conversely, stable, nutrient-rich laboratory cultures seem to favour complete denitrifiers, which maximise energy generation through denitrification. To resolve the ecological significance of metabolic trade-offs in denitrifying microbiomes, we advocate for mechanistic studies that integrate mixed-culture enrichments mimicking natural environments, multi-meta-omics, and targeted physiological characterisations. These undertakings will greatly advance our understanding of global nitrogen turnover and nitrogenous greenhouse gases emissions.

## Division of labour in microbial ecosystems

Labour division is ubiquitous in complex communities, from human societies to bee and ant colonies [1], multicellular organisms [2], and microbial ecosystems [3]. Microbial communities benefit from dividing long and complex biochemical pathways among different species. Shorter pathways require less enzymes, allowing individual cells to allocate more of their limited resources (e.g. energy, elemental building blocks, synthesis machinery) and cellular space (e.g. cytoplasm, membrane, and periplasm) to energy generation, cell growth, and metabolic adaptation [4]. Division of labour can also improve overall community function by facilitating complex substrate degradation, like carbohydrates in the human gut microbiome [5]; and by minimising the impacts of inhibitory intermediates such as hydrogen, which is cross-fed between secondary fermenters and hydrogenotrophic methanogens in anaerobic environments [6]. At the same time, shorter catabolic pathways yield less energy per unit of substrate metabolised, and cellular fitness may suffer from the reduced metabolic flexibility in dynamic environments where substrate availability fluctuates [7]. Division of labour may also result in inter-species competition for nutrients and space, and impact reaction efficiency due to the additional requirement of cross-membrane metabolite transport [8]. Trade-offs between growth, energy generation, and metabolic flexibility drive the assembly and function of any microbiome occupying a specific environmental niche [9].

Among global biogeochemical cycles, the network of nitrogen transforming organisms builds on modular metabolic pathways. Nitrification, the oxidation of ammonia (NH_3_) via nitrite (NO_2_^−^) to nitrate (NO_3_^−^), can be performed in two steps by separate guilds (ammonia oxidising bacteria or archaea—AOB or AOA—and nitrite oxidising bacteria—NOB) or by a single organism (complete ammonia oxidising—comammox—bacteria) [10]. Comammox was first theoretically proposed based on a trade-off between growth rate and biomass yield [7], and was experimentally confirmed a decade later with enrichments from slow-growing (low nutrient flux) oligotrophic (low nutrient levels) biofilm systems [11, 12]. The produced nitrate is reduced to nitrite, which can be reduced back to ammonia via dissimilatory nitrite reduction to ammonia (DNRA), or successively denitrified to gaseous nitric oxide (NO), nitrous oxide (N_2_O) and dinitrogen gas (N_2_). DNRA and denitrification thus compete for the same electron acceptor (nitrate or nitrite) and donor (commonly organic carbon) [13]. Denitrification can be catalysed by complete denitrifiers performing all reduction steps, or partial denitrifiers that divide the labour by performing only one or few reduction steps [10]. Complete and partial denitrifiers compete for the same substrates (organic carbon and nitrogen oxides), yet they occupy different ecological niches due to their distinct ecophysiological traits [14–17]. Most denitrifiers are facultative aerobes, mainly denitrifying under anoxic conditions, as commonly found in soil and ocean depths [10, 18]. Despite their pivotal role in global nitrogen turnover, biotechnological applications, and greenhouse gas emissions [10], the factors controlling the dominance of complete or partial denitrifiers remain elusive, hindering the understanding and management of natural and engineered nitrogen-transforming microbiomes. In this work, we focus on the catabolic modularity of the denitrification pathway, and its ecological significance. We discuss the ecological drivers selecting for complete and partial denitrifiers by critically exploring current labour division theories, from individual enzyme properties and resource allocation to rate-yield trade-offs, intermediate toxicity, and substrate limitation, and by integrating the most recent experimental and theoretical insights.

### Bridging species physiology to ecosystem phenotype

Denitrification has been studied for over a century [19], and our physiological and biochemical knowledge builds primarily on pure cultures of complete denitrifiers, particularly model organisms like *Paracoccus denitrificans* and *Pseudomonas stutzeri* [20]. Model denitrifiers not only allowed identifying the nitrate transporter and determining the cytoplasmic orientation of the nitrate reductase [21], but also led to the first identification of a nitric oxide reductase [22, 23] and the inhibitory role of oxygen [24]. Additionally, the electron transport chain of *Paracoccus denitrificans* has been used as the basis for the general biochemical architecture of the denitrification respiratory network [20]. Yet, *P. denitrificans* and *P. stutzeri* hardly dominate natural and engineered ecosystems [25–28], and denitrifiers are biochemically, physiologically, and ecologically very diverse, as exemplified by the existence of partial and aerobically denitrifying organisms [17, 18, 29]. Insights gained from complete denitrifying model organisms can thus not be extrapolated to all denitrifiers, and their generalisability to more complex ecosystems remains limited. The historical focus on complete denitrifiers likely results from the enrichment and isolation methods used in the past: nitrate was provided as the sole substrate, restricting the isolation to nitrate reducers; and the production of bubbles was often used as a denitrification selection criterium, excluding single-step nitrate reducers [17, 30]. Moreover, partial denitrifiers accumulating toxic denitrification intermediates (e.g. NO) as end products are unlikely to survive in pure cultures [17]. This limitation has recently been addressed through the development of new protocols, resulting in the isolation and characterisation of 61 partial denitrifiers from soil [17]. These advances hold promise to dramatically expand our repertoire of physiologically characterised partial denitrifiers. However, cultivation methods alone cannot capture the full breadth of denitrifying organisms nor resolve their ecological role, highlighting the complementary need for more *in situ* ecological studies.

**Figure 1:**
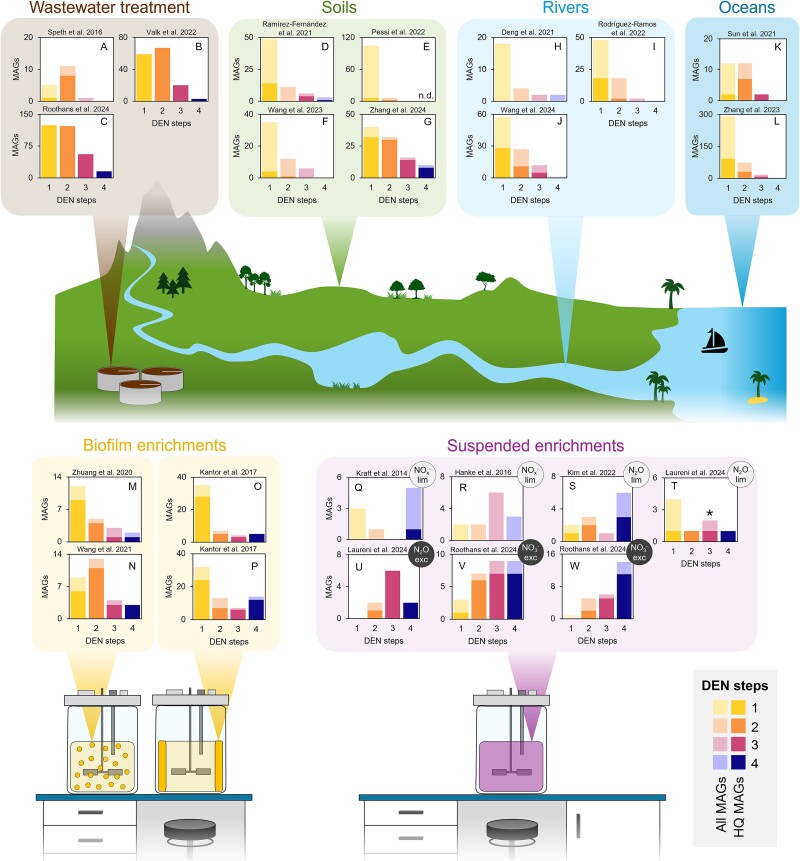
**Summary of the distribution of complete and partial denitrifiers from 1571 published metagenome-assembled genomes: Dynamic engineered and natural environments (top), and laboratory experiments (bottom).** Each graph represents the number of MAGs containing genes encoding the catalytic subunit of 1, 2, 3, or 4 denitrification steps (*narG/Z*, *napA*, *nirK*, *nirS*, *norZ*, *norB*, *nosZ*). The HQ MAGs in each study are highlighted with darker colours. The dynamic environments included (i) wastewater treatment plants: full-scale partial nitritation-anammox (A [38]) and two activated sludge systems (B [27, 37], C [39]; 83 MAGs are the same in these two studies); (ii) soils: coastal soil (D [31]), heathlands and meadows (E [25]—did not report the presence or absence of *nar*/*nap* in the MAGs), wetland (F [33]), and agricultural soil (G [32]); (ii) rivers: sediments and surface water (H [34]), and sediments (I [35], J [36]); (iii) oceans: oxygen deficient zones (K [26], L [15]). The biofilm laboratory enrichments included two granular reactors performing anammox (M [40]) and phosphate removal (N [41]), and two biofilms growing on reactor walls removing ammonium sulphate (O) and thiocyanate (P) [42] (34 MAGs are the same in these two reactors). The continuous suspended laboratory enrichment cultures include two supplied with limiting nitrate and nitrite (Q [43], R [44]), two with limiting N_2_O (S [45], T [46]), one with excess N_2_O (U [46]), and two with excess nitrate (V, W [29]). *where available, the distribution of MAGs in terms of relative abundance profiles was similar to the frequency profiles represented here [15, 26, 29, 39, 40, 46]; culture T was the only exception as it was dominated by a single 3-step denitrifier HQ MAG [46] (Supplementary Table S2). The quality and functional annotations (i.e. the identification of the catalytic subunit of the genes of interest) of all MAGs were taken from the corresponding literature studies.

**Figure 2:**
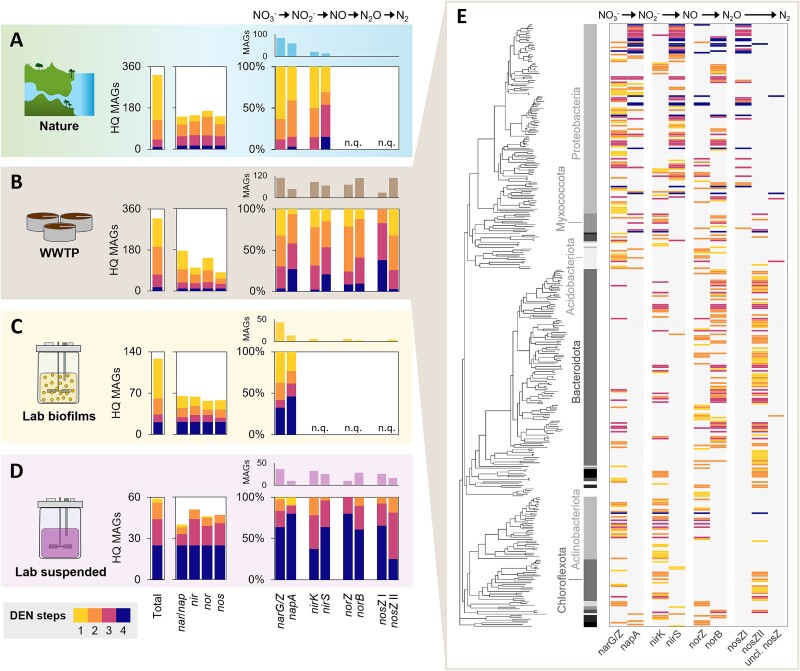
**Denitrification gene partitioning in natural and engineered ecosystems.** The colours from light to dark represent denitrifiers with genes encoding 1, 2, 3, or 4 denitrification steps. **(A-D)** The left bar charts represent the total number of unique denitrifying HQ MAGs recovered from natural environments (A, including soils [31, 33], rivers [35, 36], and oceans [15]), wastewater treatment plants (B [39], taken as representative considering that over half of the MAGs from [37] overlapped with this study), laboratory biofilms (C [40, 42]) and suspended cultures (D [29, 43, 45, 46]), and the number of HQ MAGs encoding each of the four denitrification steps (*nar*/*nap*, *nir*, *nor*, *nos*). On the right, the total amount (top) and proportion of one-, two-, three-, and four-step denitrifying HQ MAGs (bottom) are distributed over the gene homologues. The quality and functional annotations (i.e. the identification of the catalytic subunit of the genes of interest) of all MAGs were taken from the corresponding literature studies. Some studies made no distinction between *nirK*/*S*, *norZ*/*B*, and *nosZ* I/II or did not recover enough HQ MAGs, so these gene distributions appear as not quantified (n.q.) in nature (A) and biofilms (C). **(E)** 349 high-quality MAGs recovered from wastewater treatment microbiomes, 14 nitrifying and 304 non-nitrifying MAGs contained at least one denitrification gene. The heatmap shows the gene distribution among complete and partial denitrifiers: cytoplasmic- (*narG, narZ*) and periplasmic nitrate reductase (*napA*); copper-based (*nirK*) and cytochrome *cd1* nitrite reductase (*nirS*); quinol- (*norZ*) and cytochrome *c*-dependent nitric oxide reductase (*norB*); nitrous oxide reductase clade I (*nosZ* I), clade II (*nosZ* II), and unclassified (uncl. *nosZ*). The MAGs are ordered according to the phylogenetic tree and the six most frequent phyla are highlighted. Data were adapted from [39].

Natural communities are inherently complex, challenging their taxonomic and functional characterisation. Rapid advancements in genome-resolved metagenomics allow now to recover near-complete draft genomes from complex microbiomes and more accurately identify organisms genetically encoding the complete or partial denitrification pathway. We analysed the genetic functional profiles of 1571 published metagenome-assembled genomes (MAGs) containing at least one denitrification gene (encoding a catalytic subunit) across ecosystems. Given the challenge of distinguishing the physiological roles of denitrification—such as energy conservation in canonical denitrifiers vs. redox balancing or detoxification in organisms like ammonia oxidisers—based solely on genomic data, our analysis includes also 24 MAGs identified as nitrifiers. Nearly 60% of all MAGs were high-quality (HQ, ≥90% complete, ≤5% contaminated) (Fig. 1; Supplementary Table S1). It is evident that labour-dividing partial denitrifiers predominate in complex environments, featuring dynamic availability of multiple substrates and high microbial diversity, and being often spatially stratified. Conversely, complete denitrifiers seem to be favoured in continuous suspended cultures characterised by stable availability of one or few substrates, homogeneity, and low microbial diversity. MAGs recovered from soils [25, 31–33], river sediments [34–36], oceanic oxygen deficient zones [15, 26] and wastewater treatment systems [27, 37–39] are mostly partial, often single-step, denitrifiers (Fig. 1). Similarly, biofilm systems, which closely resemble natural environments in their complexity, stratification, and metabolic diversity, were dominated by organisms encoding one or two denitrification steps [40–42] (Fig. 1). In contrast, continuous suspended laboratory cultures predominantly selected for denitrifiers with genes encoding three or four steps [29, 43–46]; the relatively lower number of recovered MAGs also reflects the limited complexity of these ecosystems (Fig. 1). Although one might argue that the reported prevalence of partial denitrifiers in complex environments is due to the challenge of recovering near-complete MAGs, the increasing number of high-quality denitrifying MAGs from natural environments [15, 32] and wastewater treatment plants [27, 39] (Figs. 1 and 2A-B) rules out methodological biases. Besides, most gene-centric metagenomic studies find unbalanced abundances of denitrification genes, with *nar* and *nor* often being the most abundant in ocean and soil microbiomes [47–49], further supporting the low frequency of complete denitrifiers in natural environments. The clear prevalence of partial denitrifiers across all studied complex environments suggests a competitive advantage of dividing labour over performing complete denitrification, yet the underlying selection principles remain unclear. Expanding the current database of HQ MAGs is paramount, specially to enable functional analyses at transcriptional and translational levels (Box 1). Nevertheless, the experimental data available to date already provide important insights to explore the ecological drivers explaining the diversity of denitrifying microbiomes.

### Denitrification gene patterns and physiological diversity

The publicly available 878 unique denitrifier HQ MAGs allow to explore associations between functionally homologous denitrification genes and denitrification pathway completeness (Fig. 2; Supplementary Table S3). Across natural environments, out of the 128 HQ MAGs with differentiated nitrate reductases, 84 contained the *narG*/*Z* gene, but none was a complete denitrifier (Fig. 2A). Almost two thirds of the HQ MAGs lacked genes for any other denitrification step (Fig. 2A). The only two complete denitrifiers contained *napA*. Similarly, most *nirK*-harbouring MAGs lacked other denitrification genes, whereas the two *napA*-harbouring complete denitrifiers contained *nirS* (Fig. 2A). The *napA*/*narGZ* and *nirS*/*K* partitioning between complete and partial denitrifiers, respectively, was observed across all environments, even if the proportion of complete denitrifiers increased from natural environments to wastewater treatment plants, biofilms, and suspended cultures (Fig. 2A-D). Another study also observed a higher occurrence of *nirS* in complete denitrifiers when comparing genomes from natural environments, wastewater, and animal and plant hosts, yet nitrate reductase homologues were not included in their analysis [16]. The functionally homologous genes *norZ/norB* and *nosZ I/nosZ II* are seldomly differentiated in metagenomic studies (Fig. 2A-D). Nevertheless, based on the wastewater treatment microbiomes and laboratory cultures (Fig. 2B and2D), *norZ* and *norB* genes appear to be equally distributed between complete and partial denitrifiers, whereas 80% of all identified complete denitrifiers harboured the *nosZ* clade I genes. All N_2_O-reducing specialists contained the *nosZ* clade II genes (Fig. 2B and2D), confirming the previously suggested higher occurrence of *nosZ* I and *nosZ* II in complete and partial denitrifiers, respectively [16]. This pattern may be associated with a potential incompatibility between nitrate and *nosZ* II expression, recently observed in a *Thauera* species [54]. Taxonomically, among the 318 denitrifying HQ MAGs we recovered from wastewater treatment microbiomes, *napA*, *nirS* and *nosZ* I, and complete denitrifiers clustered predominantly within the *Proteobacteria* phylum (Fig. 2E). This taxonomic clustering aligns with prior findings [16, 55–57], and suggests that evolutionary and physiological mechanisms shape denitrification gene patterns. The physiological difference between the nitrate reductases is relatively well established, with Nap-expressing cells often featuring higher nitrate affinities and Nar providing more energy and a potentially faster turnover of nitrate (Box 2) [47, 56, 58, 59], yet this is currently not the case for the other reductases. NirK, taxonomically more disperse [57] and less resource demanding to produce than NirS [60], has been hypothesised to provide a higher physiological adaptability in dynamic environments [57, 61], yet this remains to be experimentally verified [57]. Both NirS and NosZ II have been hypothesised to have a higher affinity for their substrates and a higher tolerance to oxygen compared to their counterparts NirK and NosZ I [60–62], yet these observations are limited to a few strains and have been contradicted by other studies [63–65].

Box 1. Denitrification genotype vs. phenotype.

The distinction between complete and partial denitrifiers is here based on currently available medium and high-quality denitrifying MAGs (Figs. 1 and 2). The presence of a gene, however, does not imply its transcription or translation under a given condition, let alone provide information on the activity of the encoded protein and the organism itself [17]. For instance, in a wastewater treatment microbiome, the *nirK* and *nirS* genes were both widespread in non-nitrifying MAGs (Fig. 2B), yet we only detected the NirS enzyme from these MAGs [39]. The expression of denitrification enzymes in model denitrifiers like *P. denitrificans* has been shown to be controlled by environmental factors – typically activated by nitrate, nitrite, and nitric oxide, and repressed by oxygen and nitric oxide – whereas the expression of different reductases is not necessarily coordinated [20, 50–52]. Complete denitrifiers may exhibit a partial denitrifying phenotype under certain conditions, or express all reductases even in the absence of their substrates. For example, all denitrification enzymes were detected in enrichments solely fed with NO or N_2_O [46, 53]. On these grounds, it is clear that the genetic fingerprint of denitrifying communities represents only the first step in resolving their assembly and function. We advocate for more studies recovering HQ MAGs from complex and cultured nitrogen-converting communities as solid reference for the integration of metatranscriptomic and metaproteomic analysis in answering ecologically focused mechanistic questions.

**Table 1:** **Proton translocation across the cell membrane for each nitrogen oxide reduction step.** The proton translocation was determined considering NADH as the electron donor, and was normalised for two electrons accepted by each enzyme complex. Denitrification and DNRA: Nar – cytoplasmic nitrate reductase; Nap – periplasmic nitrate reductase. Denitrification: Nir – copper or cytochrome *cd1* nitrite reductase; *c*Nor – cytochrome *c*-type nitric oxide reductase; qNor – quinol-dependent nitric oxide reductase; Nos – nitrous oxide reductase. DNRA: Nrf – cytochrome *c* nitrite reductase. In each nitrogen oxide reduction step, protons are translocated during the oxidation of NADH and the quinol pool. The total proton translocation (ΔH^+^) of each step and the standard potential (E^0^) of each redox pair are presented. [18, 20, 79]

			**NADH oxidation (ΔH** ^**+**^ **/2 e** ^**−**^ **)**	**Quinol pool oxidation (ΔH** ^**+**^ **/2 e** ^**−**^ **)**						
**Redox reaction** **(2 e**^**−**^ **transfer)**	**E** ^**0**^ **(V)**	**Enzyme complex**	**NADH dehydrogenase (complex I)**	**Cyt *bc1* (complex III)**	**Nar**	**Nap**	**qNor**	**Nrf**	**Total** **ΔH**^**+**^**/2e**^**−**^	**Total** **ΔH**^**+**^**/N**
NO_3_^−^ ➔ NO_2_^−^	+0.43	Nar	4	-	2	-	-	-	6	6
		Nap	4	-	-	0	-	-	4	4
2 NO_2_^−^ ➔ 2 NO	+0.36	Nir	4	2	-	-	-	-	6	3
2 NO ➔ N_2_O	+1.18	*c*Nor	4	2	-	-	-	-	6	3
		qNor	4	-	-	-	0	-	4	2
N_2_O ➔ N_2_	+1.36	Nos	4	2	-	-	-	-	6	3
1/3 NO_2_^−^ ➔ 1/3 NH_4_^+^	+0.34	Nrf	4	-	-	-	-	0	4	12

Beyond individual enzymes, a physiological yield-rate trade-off has been proposed to govern the competition between generalists (complete denitrifiers) and specialists (partial denitrifiers) [66], so it is here also discussed in analogy to nitrification [7]. As facultative aerobes, denitrifiers normally generate energy by oxidising organic carbon and reducing nitrogen oxides in the absence of (sufficient) oxygen. Complete denitrification effectively yields more energy, i.e. generates more proton motive force, per mole of nitrogen oxides than partial denitrification (Box 2). Complete denitrifiers are thus expected to feature higher biomass yields, in analogy to the higher yield of comammox on ammonia [7]. In turn, a shorter catabolic pathway allows partial denitrifiers to increase the concentration of each denitrification reductase, increasing the ATP production rate and potentially resulting in higher growth rates like AOB [4, 7]. On these grounds, with a higher growth yield on nitrogen oxides and lower maximum growth rates, one would expect complete denitrifiers to dominate slower-growing, nitrogen-limited systems, where an efficient use of available resources is more beneficial than faster growth [67]. Yet, complete denitrifiers appear to dominate faster-growing well-mixed continuous suspended laboratory cultures [29, 43, 53], whereas they are outnumbered by partial denitrifiers in most slower-growing ecosystems including oceans [15, 26], freshwater systems [34–36], soils [25, 32, 33], wastewater treatment plants [27, 38, 39], and laboratory biofilm systems [40–42] (Fig. 1). Organic carbon is often limiting in these complex environments [14, 35, 68, 69], so the dominance of partial denitrifiers could simply be determined by the limiting substrate (nitrogen oxides or organic carbon) (Box 2), as recently proposed by a theoretical modelling study [14]. However, the available experimental evidence is limited and seems to refute this hypothesis, as two carbon-limited laboratory cultures enriched for complete denitrifiers (Fig. 1V-W) [29] and a largely nitrogen-limited soil enriched for partial denitrifiers (Fig. 1E) [25]. Additionally, DNRA-performing bacteria have been shown to outcompete denitrifiers in nitrogen-limited laboratory cultures and soils [13, 70, 71], potentially further narrowing the ecological niche for complete denitrifiers. This may also underly the higher frequency of DNRA-performing bacteria over complete denitrifiers in most environments, e.g. 51 and 17 DNRA vs. 6 and 0 complete denitrifier MAGs in wastewater treatment [39] and ocean microbiomes [15], respectively. Nevertheless, other factors – such as generation time, nitrite/nitrate ratio, and type of carbon source – affect the competition between DNRA and denitrification [13, 72], and may also influence the prevalence of partial over complete denitrifiers. Future experiments are needed to confirm the validity of this observation across different conditions and multiple organic substrates. An alternative hypothesis considers the benefit for complete denitrifiers of minimising the accumulation of toxic intermediates, such as free nitrous acid (HNO_2_) disrupting the transmembrane proton gradient [73, 74]; nitric oxide potentially inactivating key enzymes [75]; and nitrous oxide inactivating vitamin B12 [76]. Though interesting, the only two studies comparing intermediate accumulation by complete and partial denitrifiers reached contradicting results. Experiments with *P. stutzeri* mutants found lower nitrite accumulation during cross-feeding [77], whereas identical experiments with *Pseudomonas aeruginosa* mutants observed lower accumulation when a single strain produced and consumed nitrite [78]. Ultimately, though further experimental confirmations are warranted, the currently available ecophysiological data do not seem to support any of the hypotheses put forward to explain the distribution of functional homologues nor the selection of microbiomes dominated by complete or partial denitrifiers.

Box 2. Denitrification bioenergetics.

The proton motive force driving ATP synthesis is generated during the transfer of electrons from a donor (e.g. organic carbon) to an acceptor (e.g. nitrogen oxides). Energy generation in denitrifying and DNRA-performing organisms depends on the availability of organic carbon and nitrogen oxides, and the configuration of the nitrogen oxide reductase modules. Apart from Nar, none of the denitrification enzymes directly contributes to proton motive force. However, for all denitrification steps, protons are translocated by NADH dehydrogenase and/or cytochrome *bc1* during the electron transport from the donor to the reductase (Table 1). Therefore, respiration with the terminal reductases Nar, Nir, cNor, or Nos translocates the same amount of protons across the cell membrane, generating an equivalent amount of ATP per electron pair transferred, despite the potentially higher thermodynamic driving force of nitric and nitrous oxide reduction (Table 1) [18, 20, 79]. Nap and qNor in denitrifiers or Nrf in DNRA-performing bacteria result in the translocation of only four protons per electron pair, instead of six (Table 1). Under carbon-limited conditions, the energy yield is constrained by the amount of electrons available, so both complete and partial denitrifiers generate the same energy (6 H^+^/2 e^−^ if Nar, Nir, cNor, and Nos are used). Conversely, in nitrogen-limited environments, complete denitrifiers and DNRA-performing bacteria are in principle favoured as they accept more electrons per mole nitrogen, which is fully reduced to dinitrogen gas or ammonia, resulting in a higher energy yield (up to 15 and 18 H^+^/NO_3_^−^, respectively). Compared to partial denitrifiers, complete denitrifiers can also benefit from using multiple nitrogen oxides as electron acceptors, and, the potential electron competition between reductases has been shown not to impact the overall electron consumption and energy generation rate in a mixed culture [80].

### Metabolic adaptability beyond respiratory flexibility

**Figure 3:**
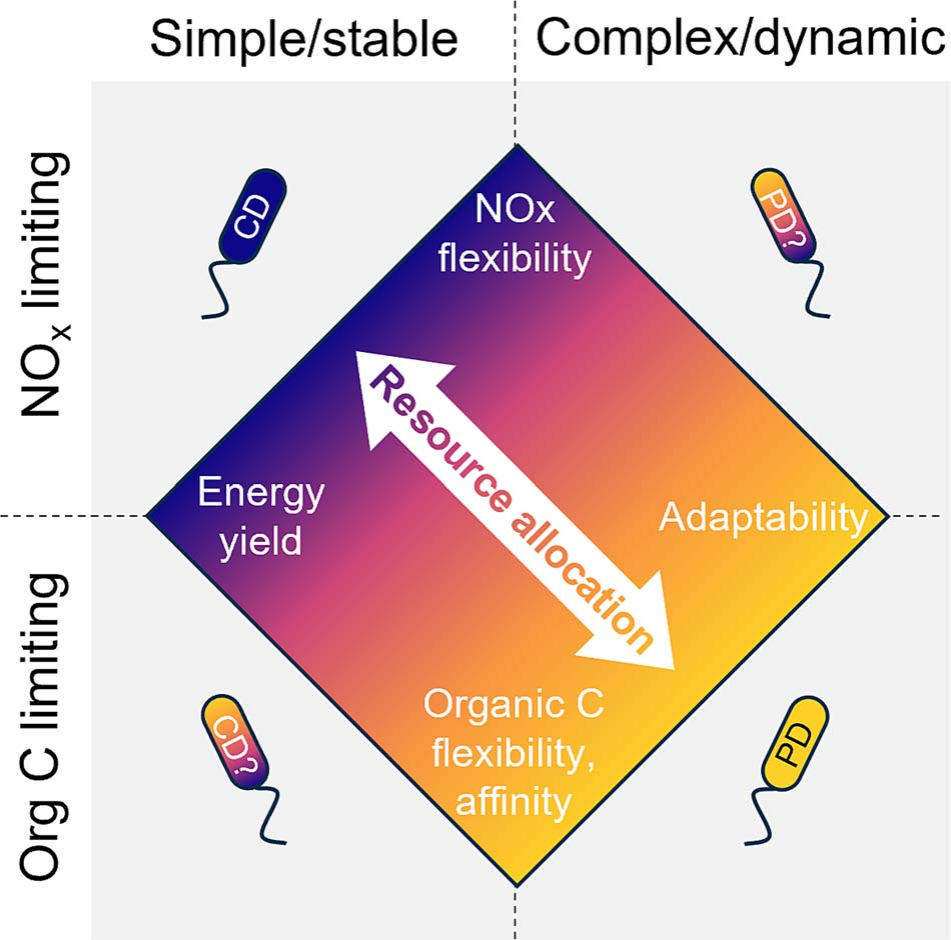
**Proposed niche partitioning drivers between complete (CD) and partial denitrifiers (PD).** Resource allocation trade-offs drive the selective advantage along two axes: environmental complexity (horizontal) and limiting substrate (vertical). Complete denitrifiers dominate nitrogen-limited stable laboratory cultures (top left) by prioritising energy yield and nitrogen oxide (NO_x_) catabolic diversity. In contrast, partial denitrifiers dominate complex environments (bottom right), which are often carbon-limited, likely due to resource allocation towards organic carbon (Org C) and other nutrient transporters, electron donor catabolic diversity, and stress response mechanisms. Nitrogen-limited complex environments (top right) and carbon-limited laboratory cultures (bottom left) remain largely unexplored, though they seem to favour partial and complete denitrifiers, respectively. Future studies should focus on these underrepresented conditions to refine this framework.

The growing evidence of partial denitrifiers being the rule rather than the exception in almost all environments cannot be explained by current labour division theories centred around catabolic pathways. Beyond the availability of nitrogen oxides and electron donors, dynamic denitrifying ecosystems experience frequent fluctuations in temperature, pH, oxygen, and nutrient levels, only to name a few environmental variables [10, 39, 81, 82]. So, we argue that resource allocation trade-offs between growth efficiency and adaptability control the selection of complete and partial denitrifiers, in analogy to other microbial systems. For example, *Escherichia coli* cells are capable of increasing substrate flexibility and affinity under substrate-limited conditions by directing resources and membrane space towards porin production [83, 84]. Slower-growing natural *Saccharomyces cerevisiae* strains switch among substrates more quickly than lab-grown strains in dynamic environments by having reduced gene regulation mechanisms, enabling the constant expression of metabolic machinery for multiple carbon sources [85]. Slower-growing bacteria are also reported to often exhibit greater antibiotic tolerance by prioritising resistance mechanisms over growth [86]. By specialising in a few denitrification steps, partial denitrifiers require fewer resources and cellular space for denitrification, which can instead be invested in the uptake of organic carbon, cofactors and micronutrients, metabolic flexibility, and stress response mechanisms, including cross-membrane transporters, alternative metabolic enzymes, and specialised RNA and protein synthesis machinery such as sigma factors and chaperones [87]. This potentially gives them a competitive advantage in complex, dynamic, oligotrophic, and carbon-limited ecosystems (Fig. 3). Conversely, in stable and nutrient-rich laboratory cultures, where metabolic flexibility and rapid adaptation requirements are reduced, complete denitrifiers likely outcompete partial denitrifiers by maximising energy generation through denitrification (Fig. 3). Metabolic trade-offs are emerging as key to explain the observed functional diversity and microbial fluctuations across ecosystems. Microorganisms in soil were proposed to excel either at growth, resource acquisition, or stress tolerance depending on environmental conditions, each contributing to the accumulation of organic matter, breakdown of complex resources through extracellular enzymes, or production of osmolytes and extracellular polymeric substances for protection [88]. Resource allocation balances have been suggested to explain patterns in stream biofilms: organisms that invest in cell adhesion and extracellular polymeric substances dominate during biofilm formation, whereas faster-growing organisms appear only in mature biofilms [89]. Similarly, a defence-growth trade-off explained the seasonal shifts in lake phytoplankton, where organisms with stronger defences but slower growth dominate during periods of increased grazing [90].

Experimentally testing how resource allocation controls the assembly and function of denitrifying microbiomes in response to environmental fluctuations remains a challenge. Recovering HQ MAGs and differentiating functionally homologous reductases is rapidly becoming the norm, and will allow to quantify and characterise complete and partial denitrifiers in complex ecosystems. Descriptive analyses of denitrifying communities are essential to continue populating the evidence on the distribution of denitrification labour division across various environments and are key to generate novel hypotheses. However, they fall short in explaining the mechanisms driving the observed patterns. We advocate for more ecologically driven mechanistic studies based on open continuous culture approaches, alongside targeted physiological and enzymatic characterisations of the growing number of denitrifiers isolated from diverse ecosystems. Non-axenic continuous enrichments allow microbial communities to evolve to a steady-state under strictly controlled operational conditions, mimicking natural environments [91]. The enriched microorganisms are, by definition, the fittest for the imposed conditions, having outcompeted all others. Combined with genome-resolved metatranscriptomic and metaproteomic analyses, mixed culture enrichments will prove essential to resolve the ecological significance of metabolic trade-offs in denitrifying microbiomes, greatly advancing our understanding of global nitrogen turnover and N_2_O emissions.

## Supplementary Material

Supplementary_Tables_wraf020

## Data Availability

No datasets were generated in this work.
